# Bone regenerative efficacy of binder-jet fabricated hydroxyapatite granules with and without biomimetic octacalcium phosphate-coated modification in a rat critical-sized calvarial defect model

**DOI:** 10.1093/rb/rbag076

**Published:** 2026-04-20

**Authors:** Jintamai Suwanprateeb, Faungchat Thammarakcharoen, Waraporn Suvannapruk, Ticomporn Luangwattanawilai, Kasem Rattanapinyopituk, Orada Sriwatananukulkit, Ruedee Hemstapat

**Affiliations:** Biofunctional Materials and Devices Research Group, National Metal and Materials Technology Center (MTEC), Pathum Thani 12120, Thailand; Thammasat University Center of Excellence in Computational Mechanics and Medical Engineering, Thammasat University, Pathum Thani 12120, Thailand; Biofunctional Materials and Devices Research Group, National Metal and Materials Technology Center (MTEC), Pathum Thani 12120, Thailand; Biofunctional Materials and Devices Research Group, National Metal and Materials Technology Center (MTEC), Pathum Thani 12120, Thailand; Department of Pharmacology, Faculty of Science, Mahidol University, Bangkok 10400, Thailand; Department of Pathology, Faculty of Veterinary Science, Chulalongkorn University, Bangkok 10330, Thailand; Department of Pharmacology, Faculty of Science, Mahidol University, Bangkok 10400, Thailand; Department of Pharmacology, Faculty of Science, Mahidol University, Bangkok 10400, Thailand

**Keywords:** hydroxyapatite, octacalcium phosphate, 3D printing, binder jetting, bone regeneration, biomimetic

## Abstract

Bone substitutes fabricated via additive manufacturing offer promising solutions for bone grafting. This study investigated the bone regeneration potential of binder-jet fabricated hydroxyapatite (HA) and biomimetic octacalcium phosphate (OCP)-coated binder-jet fabricated HA (HA-B) granules in a rat critical-sized calvarial defect model. Bone graft in the form of granules was produced via binder jet 3D printing combined with a phase transformation process and implanted into 5 mm defects for 4 and 12 weeks, with empty defects as controls. New bone formation, mechanical integration and cellular responses were assessed using micro-computed tomography, histology, histomorphometry, push-out testing and immunohistochemistry. Both HA and HA-B supported bone formation without local or systemic adverse effects. Osteoblast, osteoclast and osteocyte counts indicated active remodeling in bone graft implanted groups. Micro-CT showed significantly higher bone volume in HA-B than HA and Empty at 12 weeks, while histomorphometry showed differences only between HA-B and Empty. HA-B also exhibited the highest push-out force, indicating superior mechanical integration. These results demonstrate that binder-jet fabricated HA and OCP-coated HA granules are safe, osteoconductive, and effective for bone repair, with OCP coating further enhancing bone formation and material-host integration.

## Graphical abstract

**Graphical Abstract:**
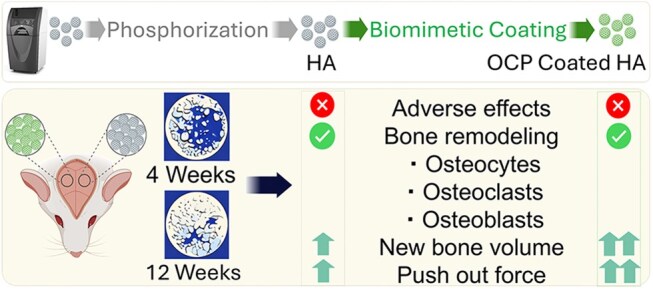

## Introduction

Additive manufacturing, or three-dimensional (3D) printing, has advanced bone grafting and tissue engineering by enabling the production of bioceramic scaffolds with tailored designs and material properties [1–7]. A variety of 3D printing techniques, including material extrusion, powder bed fusion, vat photopolymerization, material jetting, binder jetting, directed energy deposition, and sheet lamination, have been applied in the fabrication of medical devices and tissue-engineered constructs [8–11]. Each technique has unique advantages and disadvantages, making the choice of technique dependent on the intended purpose and the desired properties of the final product. Binder jetting, in particular, builds objects by selectively depositing a liquid binder onto successive layers of powder at room temperature, allowing a variety of powders to be processed. This technique eliminates the need for support structures and offers high printing speeds. Nevertheless, binder jetting is associated with several challenges, including lower mechanical strength of the produced parts, susceptibility to distortion during densification, higher surface roughness and limited printing resolution.

Despite these limitations, multiple studies have demonstrated the viability of binder jetting for manufacturing calcium phosphate scaffolds [12–17]. In our previous studies, binder jetting using calcium sulfate-based powders combined with water-based binders, followed by phase transformation, was employed to produce various calcium phosphate phases [18–22]. While some regenerative strategies utilize permanent resorbable binders to create ceramic-polymer composites such as PEVAV/β-TCP systems [23], polyvinyl alcohol (PVA) is a common component in binders used in typical binder jetting systems due to its excellent printability, film-forming ability, and biocompatibility [24–27]. Furthermore, small amounts of water-soluble polymers are often incorporated into the calcium sulfate-based powders to aid consolidation. In our work, we employed a temporary, water-soluble binder system (pregelatinized starch and commercial Zb 7). Although the exact composition of the commercial aqueous binder (Zb 7) is proprietary, it typically consists of nonhazardous components such as water, water-soluble polymers (possibly PVA), a humectant (e.g. 2-pyrrolidinone), and additives [28, 29]. Since these components are water-soluble, they are effectively eliminated from the samples during the phase conversion and subsequent washing cycles. This ensures that the final implanted granules are composed of pure binder-jet fabricated HA, as confirmed by previous characterization [30], thereby removing any potential for binder-related interference with bone regeneration and long-term concerns regarding binder biocompatibility or degradation kinetics *in vivo*. The present study builds upon this prior work and focuses on *in vivo* evaluation of bone regenerative performance using hydroxyapatite and biomimetic octacalcium phosphate-modified materials in a rat critical-sized calvarial defect model. The calcium phosphate materials fabricated via this method offer unique properties that make them differ from typical high-temperature sintering processes, including high porosity, small crystals, low crystallinity, liquid wicking ability, and biocompatibility. One of the calcium phosphates that has been investigated was hydroxyapatite (HA), which has a chemical structure that closely resembles the mineral component of natural bone. While binder jetting techniques have facilitated the fabrication of 3D-printed HA with enhanced resorbability compared to traditional high-temperature sintering methods [31, 32], the resorption of binder-jet fabricated HA remains relatively low when compared to more resorbable calcium phosphate materials.

Octacalcium phosphate (OCP), a calcium phosphate material chemically similar to the natural precursor of biological apatite, has shown superior osteoinductive and osteoconductive properties compared to other calcium phosphate biomaterials such as HA and β-tricalcium phosphate (β-TCP) [33, 34]. OCP promotes osteoblast differentiation, enhances alkaline phosphatase activity, and facilitates early and robust new bone formation *in vivo*, likely due to its unique crystal structure and surface chemistry that favor hydrolysis into hydroxyapatite and the release of bioactive calcium and phosphate ions [34, 35]. Furthermore, OCP exhibits faster resorbability than HA, which generates a dynamic microenvironment that facilitates bone remodeling through the release of calcium and phosphate ions that activate osteogenic and angiogenic signaling pathways [35–37] and also modulates the local immune environment by promoting macrophage polarization toward pro-regenerative phenotypes, indirectly supporting bone regeneration [33]. These biochemical and cellular interactions underlie OCP’s potent osteoinductivity, making it a promising bone graft substitute for regenerative applications.

Besides its application as a bone graft material, OCP coatings have been investigated as a strategy to enhance the bioactivity of various biomaterial substrates. However, the reported outcomes remain inconsistent regarding whether OCP coatings can significantly improve bone regeneration. For example, OCP coatings applied to osteoconductive materials such as titanium alloy (Ti6Al4V), HA, and biphasic calcium phosphate (BCP) have been reported to stimulate osteoinductive responses, including ectopic bone formation and enhanced bone regeneration following transcortical and intramuscular implantation in goats [38–40]. In contrast, biomimetically deposited HA/OCP coatings on titanium implants resulted in significantly lower bone-to-implant contact (BIC) and bone area compared with electrochemically deposited HA coatings in rabbit tibiae [41]. Similarly, OCP-coated xenografts evaluated in a rat calvarial defect model did not demonstrate a statistically significant improvement in bone formation compared with the uncoated xenograft [42]. These discrepancies suggest that the biological performance of OCP coatings may depend strongly on the substrate properties, coating technique, and resulting surface morphology.

In the present study, a biomimetic deposition process was performed to generate a uniform layer of leaf-like OCP crystals on the surface of binder-jet fabricated hydroxyapatite granules under mild aqueous conditions. Importantly, the coating process was applied to granules possessing an intrinsic porous microstructure produced by binder-jet printing, while preserving this porous architecture after surface modification. This feature distinguishes the present approach from many previous OCP coating studies that were performed on dense substrates such as titanium or conventional calcium phosphate ceramics. Moreover, the biomimetic method avoids high temperatures or electrochemical treatments commonly used in conventional coating techniques, thereby maintaining the structural integrity of the printed scaffold while introducing a bioactive surface chemistry.

Previously, binder-jet fabricated hydroxyapatite (HA) and biomimetic OCP-coated binder-jet fabricated HA (HA-B) were shown to provide a favorable microenvironment for human bone marrow- and umbilical cord-derived stem cell attachment, proliferation, and osteogenic differentiation. Both materials supported the upregulation of osteogenic markers and mineralized matrix deposition, while the biomimetic OCP coating further enhanced osteogenic differentiation *in vitro* [43]. However, *in vitro* studies cannot fully replicate the complexity of living systems, which influence bone regeneration outcomes. Therefore, the primary objective of this study was to evaluate the *in vivo* bone regenerative capacity of HA and HA-B using a critical-sized rat calvarial defect model. The secondary objective was to determine whether the biomimetic OCP coating enhances new bone formation and integration with host tissue compared with uncoated binder-jet fabricated HA granules. Bone regeneration was assessed using histological analysis, micro-computed tomography (micro-CT), and immunohistochemical evaluation of bone remodeling. Through this systematic *in vivo* investigation, the study aims to evaluate the biological effects of biomimetic OCP surface modification on porous binder-jet fabricated HA and to further advance their potential application in clinical bone repair.

## Materials and methods

### Sample preparation

Calcium sulfate hemihydrate powder (Lafarge Prestia Co., Ltd, Thailand) and pregelatinized starch (Thaiwah Public Co., Ltd, Thailand) were well mixed and then loaded into a binder jet 3D printer (ProJet 160, 3D Systems, Rock Hill, SC, USA) to print the spherical granules with 0.9 mm in diameter using a commercial liquid binder (Zb 7, Z Corporation, USA) that adhered the powder and solidified through evaporation, without requiring any curing. No post-processing was performed. The printing layer thickness was set at 0.1 mm. The use of a granular form was an intentional design choice for this study. Although binder jetting is capable of producing monolithic or defect-matched scaffold geometries, it was utilized here as a manufacturing method to produce uniform HA granules with controlled size, chemistry and microstructure, which are difficult to achieve using conventional fabrication methods. This particulate configuration facilitates uniform defect filling and improves experimental reproducibility across groups, while also reflecting common clinical bone grafting practices in which granular materials are widely used due to their adaptability to irregular defect geometries. In addition, the use of granules allowed evaluation of the intrinsic bioactivity of the fabricated material independent of scaffold geometry. The binder-jet printed calcium sulfate granules were then transformed to 3D-synthesized HA (referred to as HA) by immersing in 1M disodium hydrogen phosphate solution (Sigma Aldrich, USA) at 80°C for 24 h [22]. After that, the specimens were cleaned with distilled water and oven-dried. The immersion in the aqueous conversion solution and subsequent distilled water cleaning steps effectively removed the binder, resulting in a binder-free final HA product as previously characterized by XRD and FTIR [30].

HA-B was fabricated by immersing the binder-jet fabricated HA granules in an accelerated calcium phosphate solution (ACS) containing 154 mM Na^+^, 201.7 mM Cl^−^, 3.87 mM Ca^2+^, and 2.32 mM HPO4^2−^; pH 7.3 at 37°C for 8 h [44]. After immersion, the coated samples were gently rinsed and dried overnight at room temperature.

**Figure 1:**
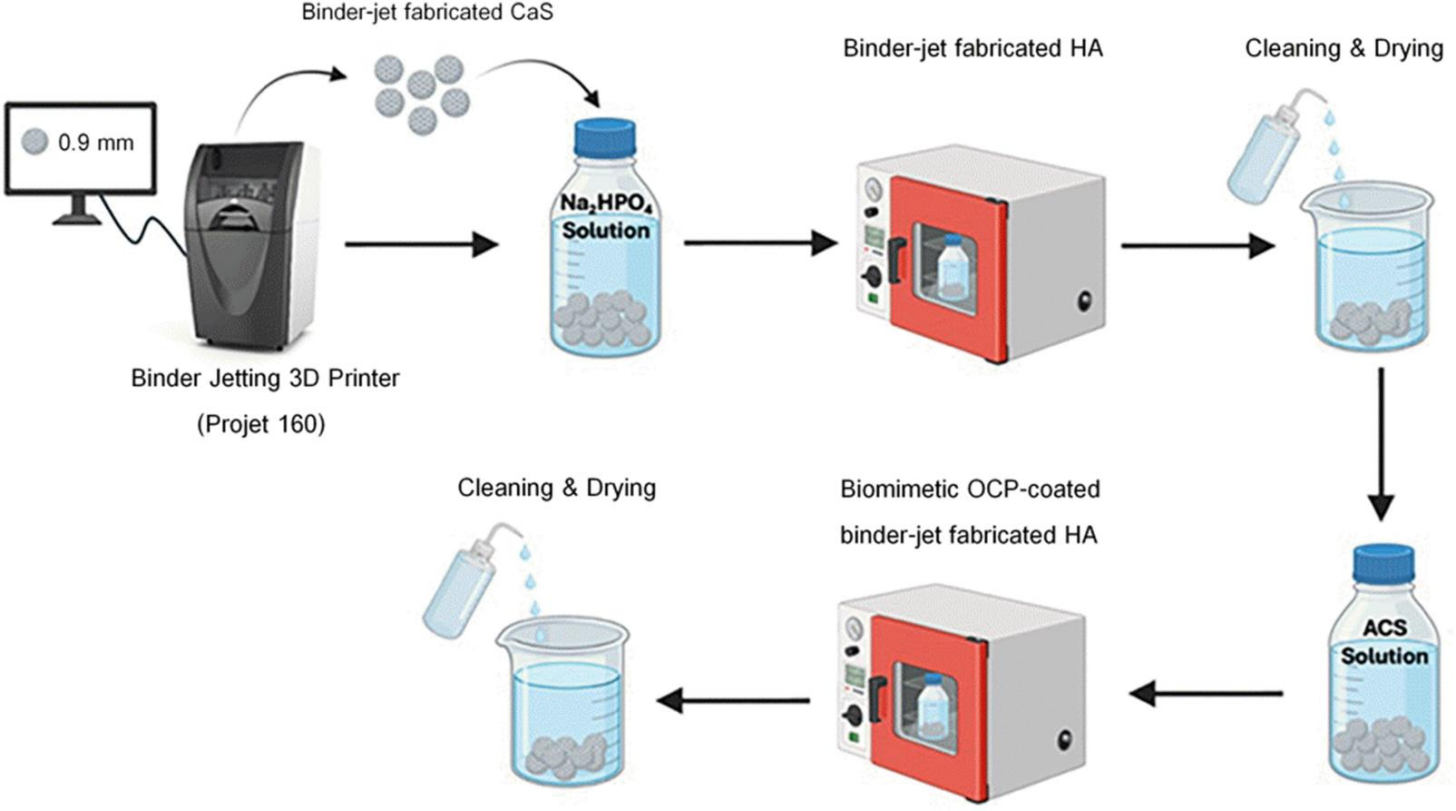
Fabrication process of binder-jet fabricated HA and biomimetic OCP-coated binder-jet fabricated HA (created with Biorender.com/Mahidol University). the binder-jet fabricated HA was produced using binder jetting followed by a phase transformation process, while the biomimetic OCP-coated binder-jet fabricated HA was obtained by immersing the binder-jet fabricated HA in an accelerated calcium phosphate solution (ACS).

The schematic diagram of the fabrication process is presented in Figure 1. All fabricated samples were packed in glass vials and enclosed in a Tyvek^®^ sterilization pouch, then subjected to ethylene oxide gas sterilization before use in the animal study.

### Animals and experimental design

All experimental procedures were approved by the Institutional Animal Care and Use Committee (IACUC) of the Faculty of Science, Mahidol University, Thailand (Approval No: MUSC58-014-329). The study was designed, conducted and reported in accordance with the ARRIVE (Animal Research: Reporting of *In Vivo* Experiments) guidelines 2.0 [45]. Male Wistar rats were obtained from the National Laboratory Animal Center, Mahidol University, Bangkok, Thailand, at 8 weeks of age. Animals were housed in pairs under standard laboratory conditions (12-h light/dark cycle, temperature 22 ± 1°C, and relative humidity 30–70%), with free access to standard commercial rodent chow and water *ad libitum*. Animals were acclimatized for 5–7 days prior to surgery, and all animals were 9 weeks old at the time of implantation. Pair housing was maintained both before and after surgery with the same cage mate to ensure social familiarity and compatibility, in accordance with established laboratory animal care guidelines [45, 46]. Animals were monitored daily throughout the post-operative period for signs of aggression, wound interference or abnormal behavior.

Thirty-six male Wistar rats (9 weeks old; body weight 310–330 g at the time of surgery) were randomly assigned to two main groups based on the designated euthanasia timepoints: 4 weeks (*n* = 18) or 12 weeks (*n* = 18) post-implantation. Within each time point, animals were further allocated into three experimental groups: Gr.-1 (Empty control; defect without implant), Gr.-2 (HA; defect implanted with binder-jet fabricated HA) and Gr.-3 (HA-B; defect implanted with biomimetic OCP-coated binder-jet fabricated HA).

Animals were randomly allocated to experimental groups using a simple randomization method by drawing lots at the individual animal level prior to surgery, without predefined stratification. Randomization was performed by a researcher not involved in the surgical procedures. Individual animals were identified using subcutaneous microchips. Allocation concealment was not implemented; however, group assignments were determined prior to surgery and were not altered throughout the study. The sample size of six rats per group per time point was selected based on consistency with previously published rat critical-size calvarial defect studies evaluating bone regeneration using micro-CT and histological analyses, in which similar group sizes were sufficient to detect biologically relevant differences [47, 48]. This sample size is widely used and validated in this model and also reflects ethical considerations to minimize animal use in accordance with the 3Rs principle.

**Figure 2:**
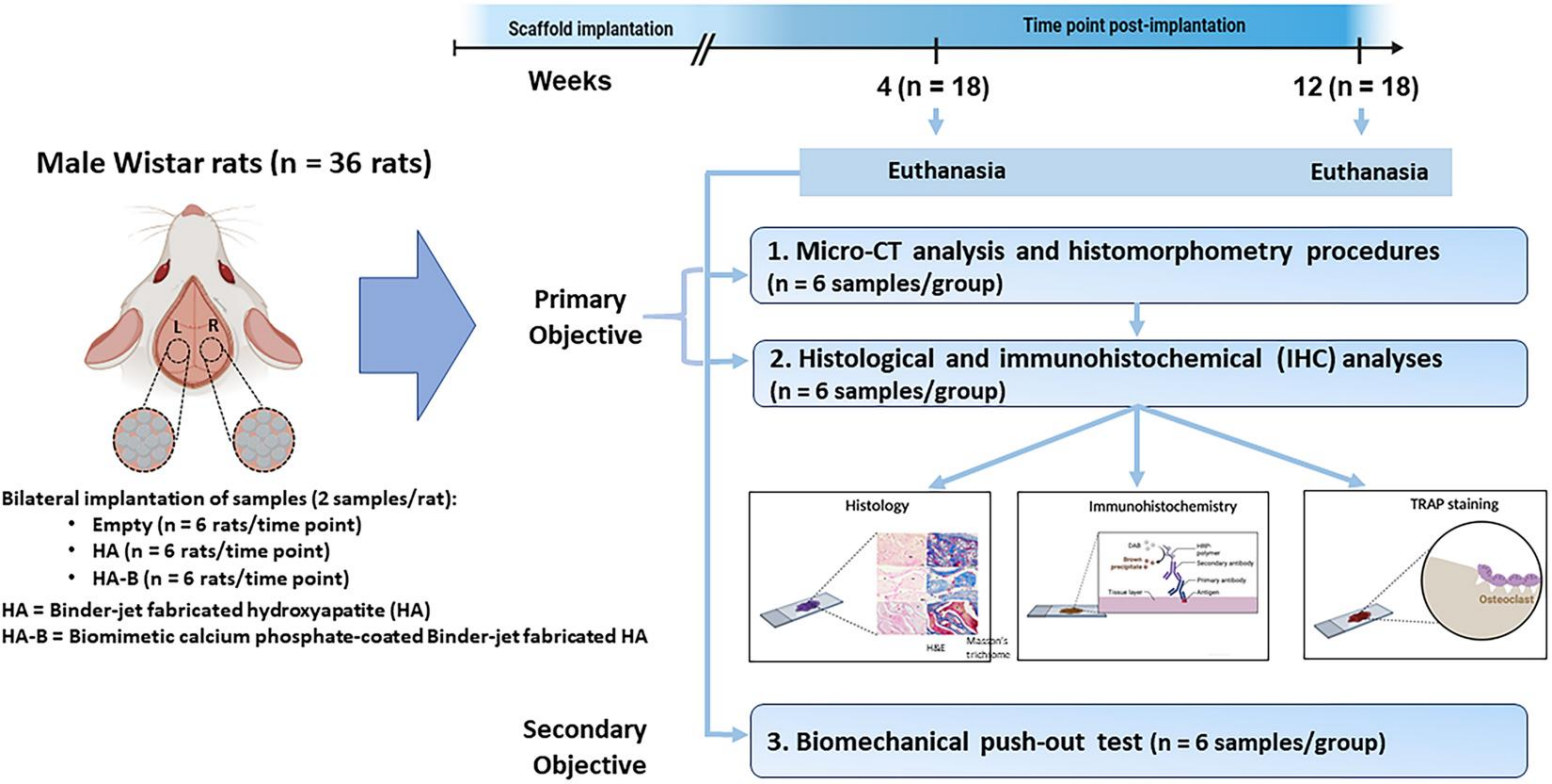
Schematic diagram summarizing the experimental design (created with Biorender.com/Mahidol University).

For implantation, each implant type was randomly assigned to the bilateral defects, ensuring that identical implants were not placed on both sides of the same animal. Animals were allowed to recover for 4 or 12 weeks, during which body weight and clinical observations were recorded pre-surgery (week 0) and weekly until the 4-week endpoint, and biweekly (weeks 2, 4, 6, 8, 10 and 12) until the 12-week endpoint, as indicators of general health. Following sacrifice, the retrieved calvarial samples (*n* = 18 per time point) were allocated into three subsets to address the study objectives. The first subset (*n* = 6) underwent non-destructive micro-CT imaging for three-dimensional quantification of bone volume, followed by processing for histomorphometric evaluation. The second subset (*n* = 6) was dedicated to histological analysis to examine tissue-material interactions and cellular response. The final subset (*n* = 6) was utilized for biomechanical push-out testing to assess the functional integration and stability of the graft (Figure 2). This sequential and partitioned allocation ensured that primary regenerative outcomes and secondary mechanical integration were evaluated independently. The final number of specimens analyzed in each group varied from the initial allocations due to technical and biological limitations. Exclusion criteria included tissue damage during harvesting, specimen loss or degradation during fixation, decalcification, or embedding, as well as misalignment or failure during push-out mechanical testing. In some cases, excessive bone overgrowth or difficulty in identifying the defect margins hindered accurate histological or mechanical evaluation. Such occurrences are common in preclinical studies and reflect the inherent challenges associated with handling and evaluating small, delicate specimens.

### Induction of calvarial bone defect model and sample implantation

**Figure 3:**
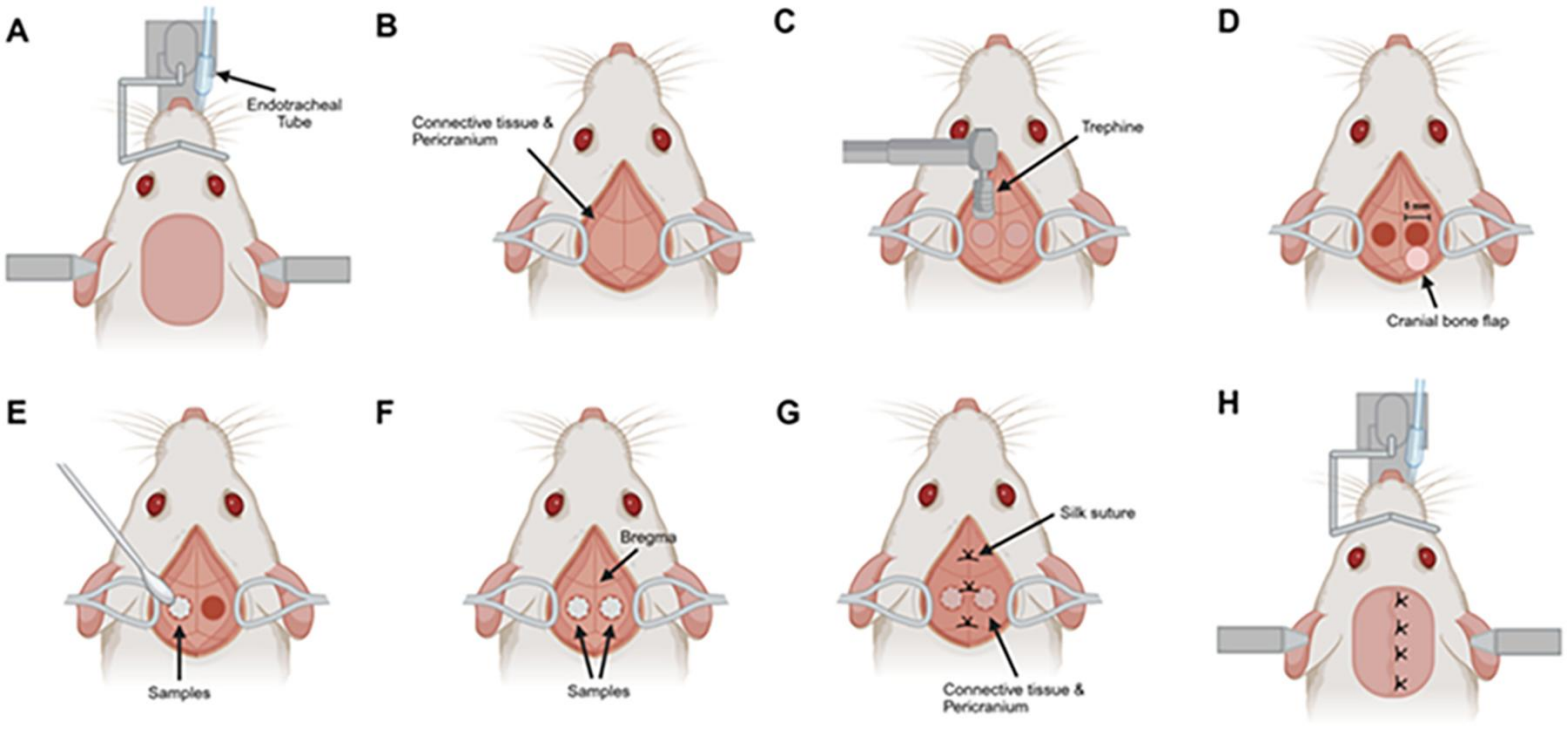
Procedure of sample implantation in a rat calvarial bone defect model. (**A**) After intubation, the rat’s head was stabilized using stereotaxic frame. (**B**) The loose connective tissue and pericranium were carefully retracted to expose the calvarium. (**C** and **D**) Bilateral critical-sized, full-thickness calvarial bone defects (5 mm in diameter) were created using a trephine drill, and the cranial bone flaps were removed. (**E** and **F**) Implant samples were placed into each defect site. (**G** and **H**) The implanted area was closed by suturing the connective tissue and pericranium to secure the implants, followed by skin closure using 4-0 non-absorbable silk sutures. (created with Biorender.com).

Rats were anesthetized using inhaled isoflurane (Attane™, Piramal Critical Care, Inc., USA), with 5% for induction followed by endotracheal intubation. The endotracheal tube was connected to an anesthesia circuit delivering 2–3% isoflurane in oxygen to maintain anesthesia. While anesthetized, the rat’s head was secured in a standard stereotaxic frame. Fur over the calvarial region was shaved, and the surgical site was disinfected with povidone-iodine followed by 70% ethanol (Figure 3A). A longitudinal midline skin incision (2–3 cm) was made posterior to the eyes, extending in a rostral- to-caudal direction. The overlying loose connective tissue and pericranium were carefully retracted to expose the calvarial bone (Figure 3B).

Bilateral critical-sized, full-thickness calvarial bone defects (5 mm in diameter) were created using a trephine drill (Saeshin Precision Co., Ltd.; Daegu, Korea) at the dorsal region of both parietal bones. The 5 mm defect is a well-established critical-size defect in a rat that does not undergo spontaneous bone regeneration and is widely used for evaluating bone regenerative efficacy [49, 50]. Care was taken to avoid perforation of the underlying meninges, and the resulting bone flaps were gently removed (Figure 3C and D). Continuous irrigation with sterile normal saline was used throughout the drilling procedure to prevent thermal osteonecrosis. Implant samples (approximately 0.01 g per defect) were placed into each defect site (Figure 3E and F). The surgical site was closed by first suturing the loose connective tissue. Subsequently, the pericranium was repositioned and re-sutured using 4-0 non-absorbable silk sutures (Ethicon^®^, Inc., Livingston, United Kingdom) (Figure 3G and H) to secure the implants and provide a natural biological barrier against soft tissue infiltration, followed by the closure of the skin incision. Representative photographs of the surgical procedures are provided in Supplementary Figure S1.

Post-operative care included subcutaneous administration of tramadol (10 mg/kg; Tramada-100®, L.B.S. Laboratory Ltd. Part., Bangkok, Thailand) twice daily for 3 days for pain management, and cefazolin (20 mg/kg; Cefaben®; L.B.S. Laboratory Ltd. Part., Bangkok, Thailand) twice daily for 5 days to prevent post-operative infection.

### Blood biochemistry analysis

Blood samples were collected from the lateral tail vein of each rat pre-operatively (baseline) and from the posterior vena cava at the study endpoints (4 or 12 weeks post-implantation) to monitor overall health status. Liver and kidney function were assessed by measuring blood biochemistry parameters, specifically alanine aminotransferase (ALT) for hepatic function and creatinine for renal function.

### Micro-CT analysis and histomorphometry procedures

At the study endpoints (4 and 12 weeks post-implantation), rats were euthanized via an intraperitoneal injection of thiopental (100 mg/kg; Anesthal^®^, Scott-Edil Pharmacia Ltd., Solan, India). Following euthanasia, the animals were decapitated, and the scalp was dissected to expose the calvarium. The cranial bones containing the implants were harvested. Three-dimensional radiographs of the skull defects were acquired post-resection using a micro-CT scanner (SCANCO Medical µCT 35, Switzerland) at an isotropic voxel resolution of 30 µm, with X-ray settings of 70 kVp and 114 μA. A hydroxyapatite calibration phantom was used to ensure accuracy. Images were segmented using a Gaussian filter, and each slice was examined to define the defect area as the region of interest. Architectural parameters were extracted from the binarized volumes of interest, including total volume (TV), bone volume (BV) and bone volume fraction (BV/TV). TV represents the full volume of the defect (a 5-mm diameter hole), BV is the volume of newly formed mineralized tissue, and BV/TV reflects the relative bone volume normalized to the total defect volume, accounting for variations in specimen thickness.

Following micro-CT, these specific samples were fixed in 10% neutral buffered formalin (Acros Organics, Geel, Belgium) for 48 h and decalcified in 10% ethylenediaminetetraacetic acid (EDTA; Kemaus, Cherrybrook, NSW, Australia) at room temperature for two weeks. After decalcification, the tissues were processed using standard histological procedures, embedded in paraffin, and sagittally sectioned at a thickness of 4 µm. The sections were stained with Masson’s trichrome (MT) and used for bone histomorphometric analysis using ImageJ software (National Institutes of Health, USA). The entire defect area was analyzed without defining a specific region of interest, allowing comprehensive assessment of bone regeneration.

### Histological and immunohistochemical (IHC) analyses

A separate subset of samples was prepared following the fixation and decalcification protocols described above. The sections were then stained with hematoxylin and eosin (H&E) to examine general bone morphology, and with Masson’s trichrome (MT) to evaluate collagen deposition and bone maturation. Histological evaluation was performed in accordance with ISO 10993-6:2016 to assess local tissue responses following implantation [51]. Hemorrhage was graded on a scale from 0 to 3 (0 = none, 3 = severe). Inflammatory response, including polymorphonuclear cells, lymphocytes, and plasma cells, was scored on a scale from 0 to 4, with higher scores indicating increasing levels of cellular infiltration from minimal presence to densely packed inflammatory cells. Foreign-body response, characterized by macrophages and multinucleated giant cell infiltration, was evaluated on a scale of 0–4, ranging from no detectable response to marked, sheet-like cellular accumulation. Fibrosis was assessed from 0 to 4, representing the progression from absence of fibrous tissue to extensive fibrous band formation. These evaluations provide a semi-quantitative assessment of local tissue response and biocompatibility to the implanted materials.

Additionally, immunohistochemical staining was performed to assess specific biological markers of bone healing. For anti-osteocalcin (OC) IHC, antigen retrieval was performed by heating tissue sections in a citrate buffer (pH 6.0), using a microwave at medium-high power for 5 min. After cooling, sections were washed with phosphate-buffered saline (PBS) (Gibco, NY, USA) and incubated with 3% hydrogen peroxide (Chem-supply, Port Adelaide, SA, Australia) in methanol (Merck, Darmstadt, Germany) for 30 min to block endogenous peroxidase activity. Nonspecific binding was blocked by incubating the sections with 1% bovine serum albumin (BSA) (EMD Millipore, Darmstadt, Germany). Sections were then washed with PBS and incubated overnight at 4°C with a mouse monoclonal anti-osteocalcin antibody ((G-5): sc-365797, Santa Cruz Biotechnology, Inc., Dallas, TX, USA) at a dilution of 1:500. After washing, sections were incubated for 45 min with a conjugated secondary antibody (Envision™, Dako, Denmark). The chromogenic reaction was visualized with a 3,3-diaminobenzidine (DAB) substrate (Abcam, Cambridge, UK), and the reaction was subsequently stopped by rinsing in distilled water. Tissue sections were then counterstained with Mayer’s hematoxylin, dehydrated through a graded ethanol series (Merck, Darmstadt, Germany), cleared in xylene (PanReac AppliChem, Darmstadt, Germany), and mounted. Rat bone tissue was used as a positive control for anti-osteocalcin expression.

Tartrate-resistant acid phosphatase (TRAP) staining was performed to identify osteoclasts. Tissue sections were incubated in a pre-warmed TRAP staining solution (Sigma-Aldrich, Steinheim, Germany) consisting of TRAP basic incubation medium (Sigma-Aldrich, Steinheim, Germany), freshly prepared naphthol AS-MX phosphate substrate (Sigma-Aldrich, Steinheim, Germany), and Fast Red Violet LB salt (Sigma-Aldrich, Steinheim, Germany). The incubation was carried out at 37°C in a water bath for 30 min, or until the control slide showed adequate staining development. Thereafter, sections were rinsed in distilled water and counterstained with 0.02% Fast Green dye for 30 s. Sections were then briefly rinsed with distilled water, dehydrated through a graded ethanol series, cleared, and mounted. TRAP-positive osteoclasts were identified by the presence of red-violet staining in the cytoplasm, contrasting with a green background. The numbers of OC-positive osteoblasts and osteocytes, as well as TRAP-positive osteoclasts, were quantified under a light microscope with NIS-Elements Analysis D software (Nikon, Japan) using three histological sections per sample at each time point.

To ensure consistency and minimize observer bias, inter-observer variability between two blinded investigators (K.R. and T.L.) was assessed before data collection. All histological slides were independently examined by both investigators, who were blinded to the sample identities. Any discrepancies in interpretation were resolved through discussion and consensus to maintain the accuracy and reliability of the data.

### Biomechanical push-out test

Push-out testing was conducted using a universal testing machine (Instron 55R4502, USA) equipped with a 10 kN load cell. The tests were carried out at a controlled temperature of 23°C and 50% relative humidity. Dissected calvarial bone samples were clamped between two acrylic plates: the upper plate with a 4.5 mm diameter hole and the lower plate with a 6.0 mm diameter hole. Each sample was carefully aligned such that the defect site was in the center of the hole in both plates. A flat-ended plunger, 4.2 mm in diameter, was attached to a load cell and used to push the material out of the defect. The plunger was applied to the center of the defect at a constant crosshead speed of 2 mm/min. The maximum force required to displace the defect content was recorded and reported as the push-out force. For the Control group, this defect content consisted of regenerated tissue, while for the implanted groups, the contents were the implant and the surrounding regenerated tissue.

### Statistical analysis

Statistical analysis and graph generation were performed using GraphPad™ Prism version 10.1.2 (GraphPad Software, Boston, MA, USA). Sample sizes were based on a previous study [49]. Data distribution was assessed using the Shapiro–Wilk test. For normally distributed data, including bone volume fraction (BV/TV; Figure 5B and C), osteoblast (at 12 weeks; Figure 9A), osteocytes (Figure 9B), and osteoclast (Figure 9C) counts, percentage of new bone and residual bone graft in the defect area (at 4 weeks; Figure 10A and C), push-out strength (Figure 11A and B), one-way ANOVA followed by Bonferroni’s multiple comparison test was used. Non-normally distributed data, such as osteoblast count (at 4 weeks; Figure 9A), and the percentage of new bone and residual bone graft in the defect area (at 12 weeks; Figure 10B and D) were analyzed using the Kruskal–Wallis test with Dunn’s post-hoc analysis. Results are presented as mean ± standard error of the mean (SEM), and statistical significance was defined as *P* < 0.05.

## Results

### Morphology and microstructure of HA and HA-B granules

**Figure 4:**
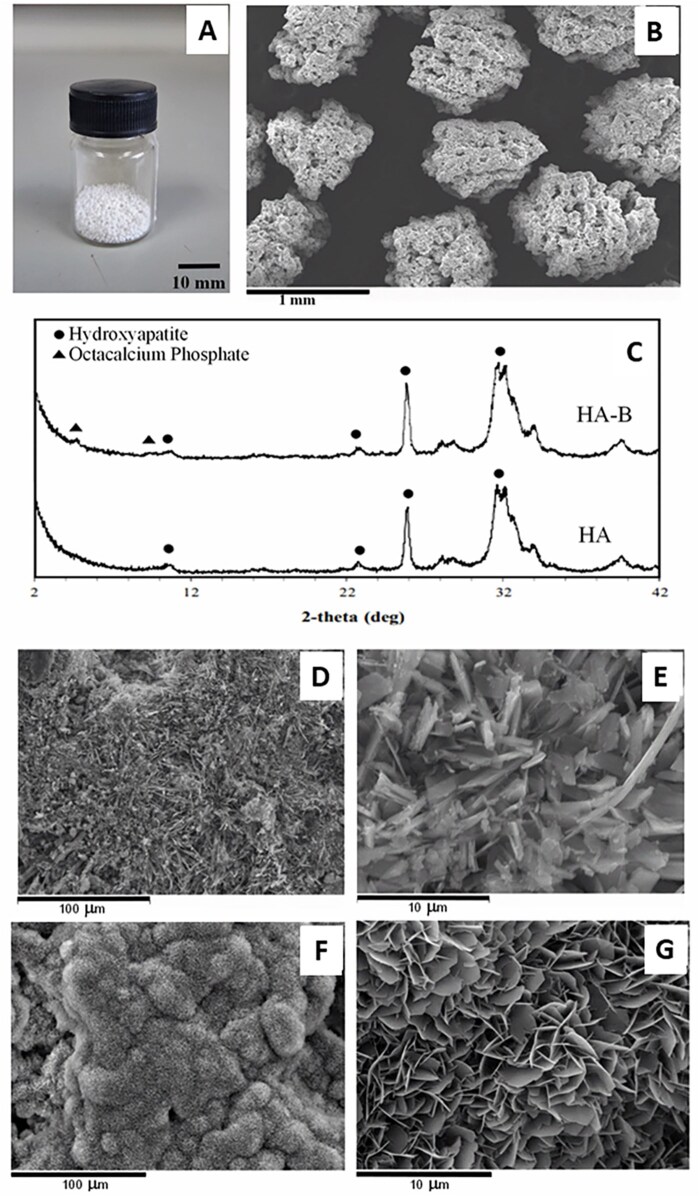
Morphology and microstructure of HA and HA-B granules utilized in this study. (**A, B**) Representative images of fabricated granules at low and high magnification, respectively (Scale bars = 10 mm and 1 mm). (**C**) XRD patterns of the HA and HA-B granules, confirming the presence of the OCP phase in the coated group (noted by the peak at 4.7 2-theta). (**D, E**) Surface microstructure of HA granules at 500× and 5000× magnification, respectively (scale bars = 100 µm and 10 µm). (**F, G**) surface microstructure of HA-B granules at 500× and 5000× magnification, respectively (Scale bars = 100 µm and 10 µm).

Figure 4A and B show the representative images of fabricated granules used for implantation. Analysis of the sampled granules confirmed a mean diameter of 0.97 ± 0.11 mm. The phase composition of both binder-jet fabricated HA and OCP-coated granules was verified via X-ray diffraction (XRD), as shown in Figure 4C. The additional characteristic peak at 2-theta of about 4.7° confirms the successful biomimetic coating of the OCP phase onto the HA phase. In accordance with the chemical and phase characterization reported in our previous studies using this fabrication method [30], the granules used in this study consisted of binder-free materials. Figure 4D–G display the morphology and microstructure of the HA and HA-B granules utilized in this study. Both samples exhibited high porosity, characterized by macropores and micropores. SEM imaging revealed a distinct shift in crystal structure from a needle-like (acicular) form with individual crystals measuring approximately 2.06 ± 0.81 µm in length in the binder-jet fabricated HA substrate (Figure 4D and E) to a porous, interlaced network of leaf-like (foliated) OCP crystals (Figure 4F and G). High-magnification analysis reveals that these individual leaves measure approximately 2.66 ± 0.48 µm in length. Due to the shared precipitation-based nature of both phases, the OCP layer is epitaxially integrated into the HA substrate, precluding the measurement of a discrete coating thickness. This observation is consistent with our previous work on biomimetic OCP coatings, where similar growth kinetics resulted in a layer thickness of approximately 4.3–8.7 µm [52]. The larger, leaf-like structures create a more open, high-aspect-ratio topography, which likely increases the specific surface area available for protein adsorption and subsequent cellular anchorage.

### General animal health and systemic safety evaluation

All animals maintained good general health throughout the study, with mean body weight (± SEM) gradually increasing in all groups at both 4 and 12 weeks post-implantation (see Supplementary Figure S2). Additionally, blood biochemistry parameters related to liver and kidney function, including alanine aminotransferase (ALT) and creatinine (Cr), respectively, remained within normal physiological ranges across all groups [53]. These results suggest that implantation of both HA and HA-B samples was safe and well tolerated *in vivo* (see Supplementary Table S1).

### Micro-CT evaluation

**Figure 5:**
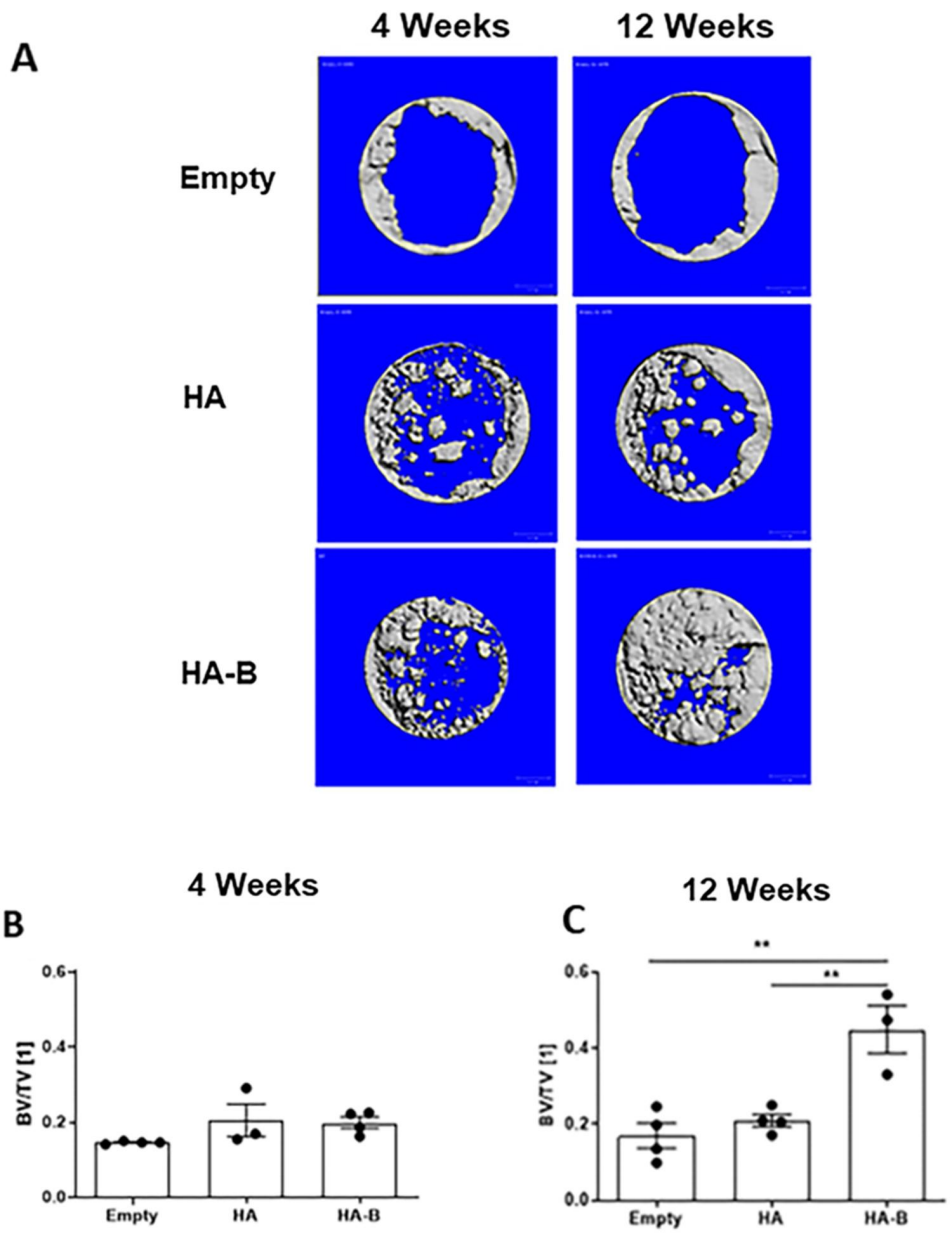
(**A**) Representative micro-CT images of calvarial bone defects at 4 and 12 weeks post-implantation. (**B, C**) Quantitative analysis of bone volume fraction (BV/TV) obtained from micro-CT at 4 weeks (**B**) and 12 weeks (**C**). *P* < 0.01 (**) were considered statistically significant.

Figure 5A shows representative micro-CT reconstructed images of skull defects from the different groups at 4 and 12 weeks post-implantation. In the Control group, bone formation was restricted to the defect margins, with the central area remaining unfilled at both time points. In contrast, both HA and HA-B groups exhibited new bone at the margins and within the central defect region at 4 and 12 weeks. At 12 weeks, the HA-B group displayed abundant regenerated bone occupying most of the defect, whereas the HA group exhibited only partial filling. Figure 5B shows the bone volume fraction (BV/TV) within the defect at 4 weeks, where both the HA and HA-B groups exhibited slightly higher BV/TV values compared to the empty group; however, these differences were not statistically significant. At 12 weeks post-implantation (Figure 5C), the HA group showed a greater BV/TV than the empty group, but this difference was not statistically significant. The HA-B group, however, demonstrated a significantly higher BV/TV compared to both the empty and HA groups (*P* < 0.01).

### Histology and histomorphometry

**Figure 6:**
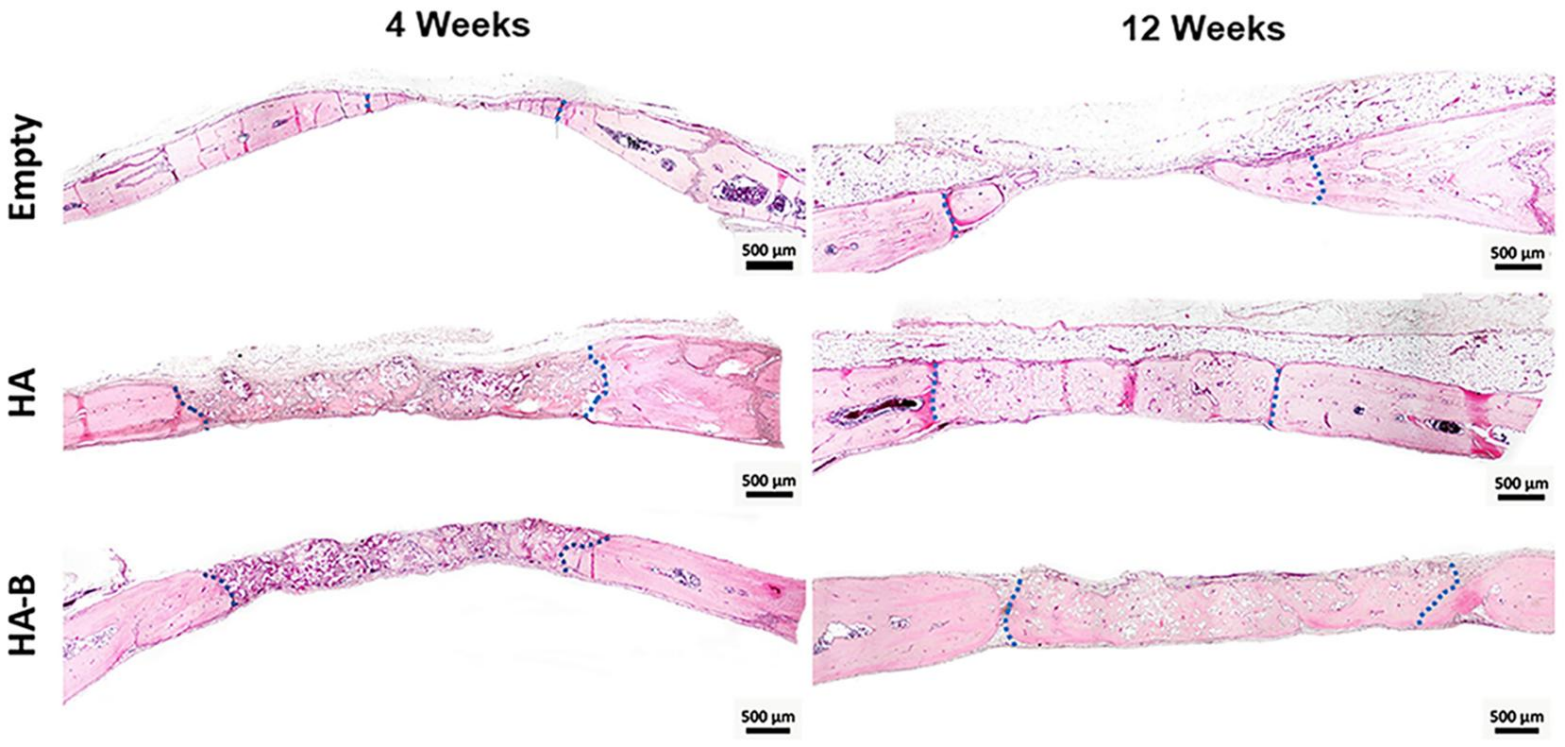
Representative histological images of calvarial bone defects at 4 and 12 weeks post-implantation, stained with H&E. The blue dashed line marks the edge of the defect. Scale bar = 500 µm.

**Figure 7:**
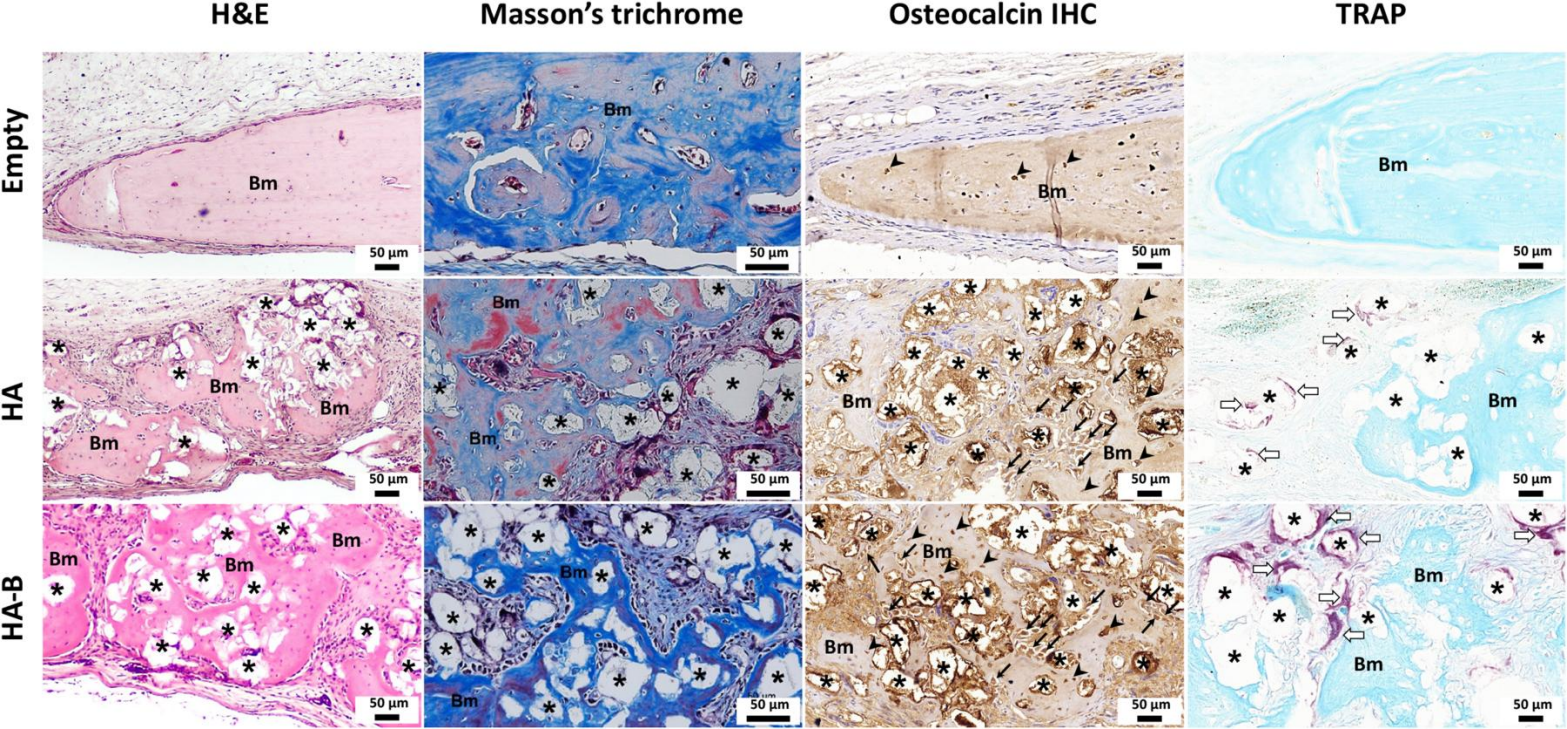
Representative histological images of calvarial bone defects at 4 weeks post-implantation, including H&E, Masson’s trichrome, osteocalcin, and TRAP staining. Bm denotes bone matrix. Asterisks (*) indicate residual implanted material. Arrows indicate OC positive osteoblasts; arrowheads indicate OC-positive osteocytes; and white arrows denote TRAP-positive osteoclasts. Scale bar = 50 µm.

**Figure 8:**
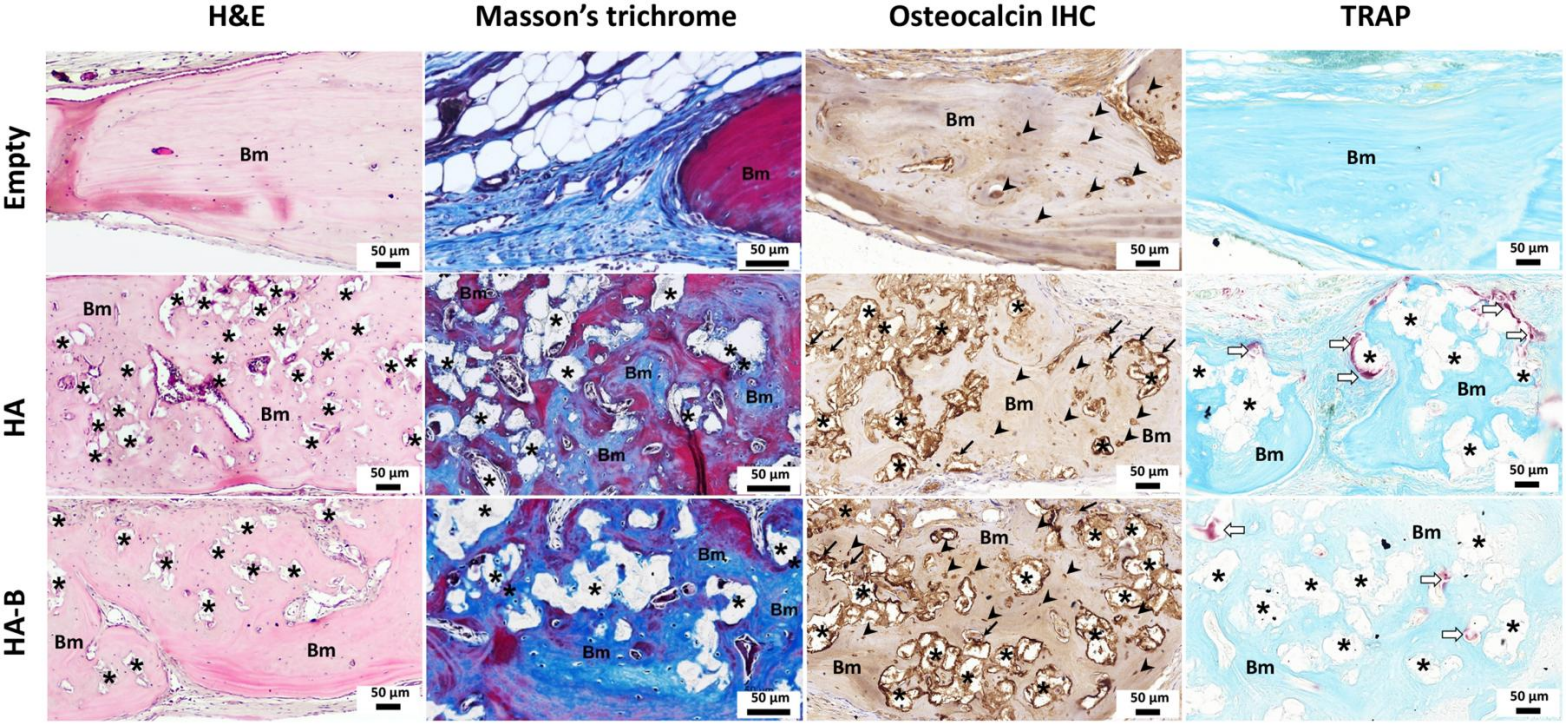
Representative histological images of calvarial bone defects at 12 weeks post-implantation, including H&E, Masson’s trichrome, osteocalcin, and TRAP staining. Bm denotes bone matrix. Asterisks (*) indicate residual implanted material. Arrows indicate OC-positive osteoblasts; arrowheads indicate OC-positive osteocytes; and white arrows denote TRAP-positive osteoclasts. Scale bar = 50 µm.

Histological evaluation using H&E staining at 4 and 12 weeks post-implantation revealed no marked tissue damage, necrosis, or excessive inflammatory infiltration in the peri-implant regions across all experimental groups (Figures 6–8). Only limited inflammatory cell infiltration, consistent with physiological wound healing, was observed. In addition, there was no indication of a marked foreign-body reaction, with only scarce multinucleated giant cell presence detected. Semi-quantitative histological scoring further confirmed these findings, with all evaluated parameters showing scores of less than 1, indicating minimal local tissue response. In the Empty group, the defect center was predominantly filled with fibrous connective tissue at both time points, with only minimal new bone formation at the defect margins (Figure 6). Notably, the defect in the Empty group appeared narrower than in the HA and HA-B groups. This difference is likely attributed to tissue processing artifacts, as the loose connective tissue in the Empty group underwent greater shrinkage during dehydration and embedding compared with the mineralized bone or granules in the HA and HA-B groups, leading to an apparent narrowing not observed in gross morphology or micro-CT images. Spontaneous bone regeneration in the Empty group remained limited even after 12 weeks. In contrast, both HA- and HA-B groups displayed substantial new bone formation at both 4 and 12 weeks, with the extent of regeneration differing according to implant type and time point (Figure 6). Masson’s trichrome (MT) staining confirmed bone matrix deposition, visible as blue and red staining areas (Figures 7 and 8), while residual implant materials were still detectable in the defect sites of all implanted groups. Immunohistochemistry staining for OC confirmed the presence of osteoblasts and osteocytes within the regenerated bone tissues (Figures 7 and 8), indicating ongoing osteogenesis. Furthermore, TRAP staining identified osteoclasts at the defect sites, reflecting active bone remodeling processes (Figures 7 and 8).

### Quantitative analysis of bone cells

**Figure 9:**
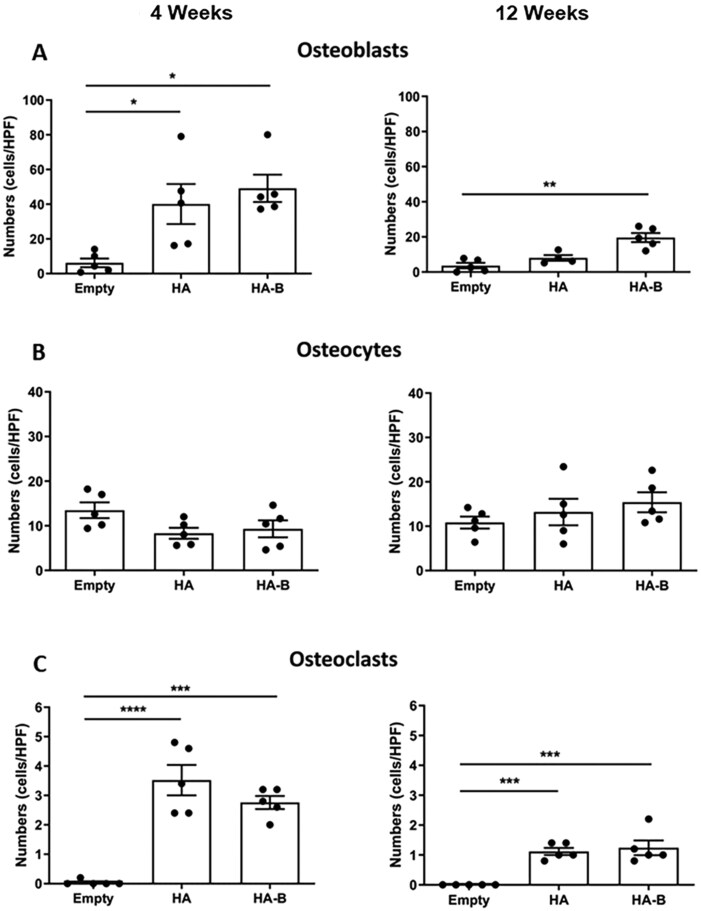
Quantification of osteoblasts, osteocytes, and osteoclasts in calvarial bone defects at 4 and 12 weeks post-implantation. Data are expressed as the number of positively stained cells per high-power field (HPF). for normally distributed data at 4 weeks (B, C) and 12 weeks (**A, B, C**), one-way ANOVA followed by Bonferroni’s multiple comparison test was used. For non-normally distributed data at 4 weeks (A), the Kruskal–Wallis test with Dunn’s post-hoc analysis was applied. *P* < 0.05 (*), *P* < 0.01 (**), *P* < 0.001 (***) and *P* < 0.0001 (****) were considered statistically significant.

At 4 weeks post-implantation, the number of OC-positive osteoblasts was significantly higher in both HA and HA-B groups compared to the Empty control group (*P* < 0.05). However, at 12 weeks, only the HA-B group maintained a significantly greater number of OC-positive osteoblasts relative to the Empty control group (*P* < 0.01), while no significant difference was observed between the HA and HA-B groups at either time point (Figure 9A). In contrast, the number of OC-positive osteocytes did not differ significantly among any of the groups at either 4 or 12 weeks post-implantation (Figure 9B). Additionally, both the HA and HA-B groups exhibited significantly higher numbers of TRAP-positive osteoclasts compared to the Empty group at both time points (*P* < 0.001 or *P* < 0.0001). No significant difference in osteoclast numbers was observed between the two implanted groups (Figure 9C).

### Histomorphometric analysis of new bone formation and residual bone graft

**Figure 10:**
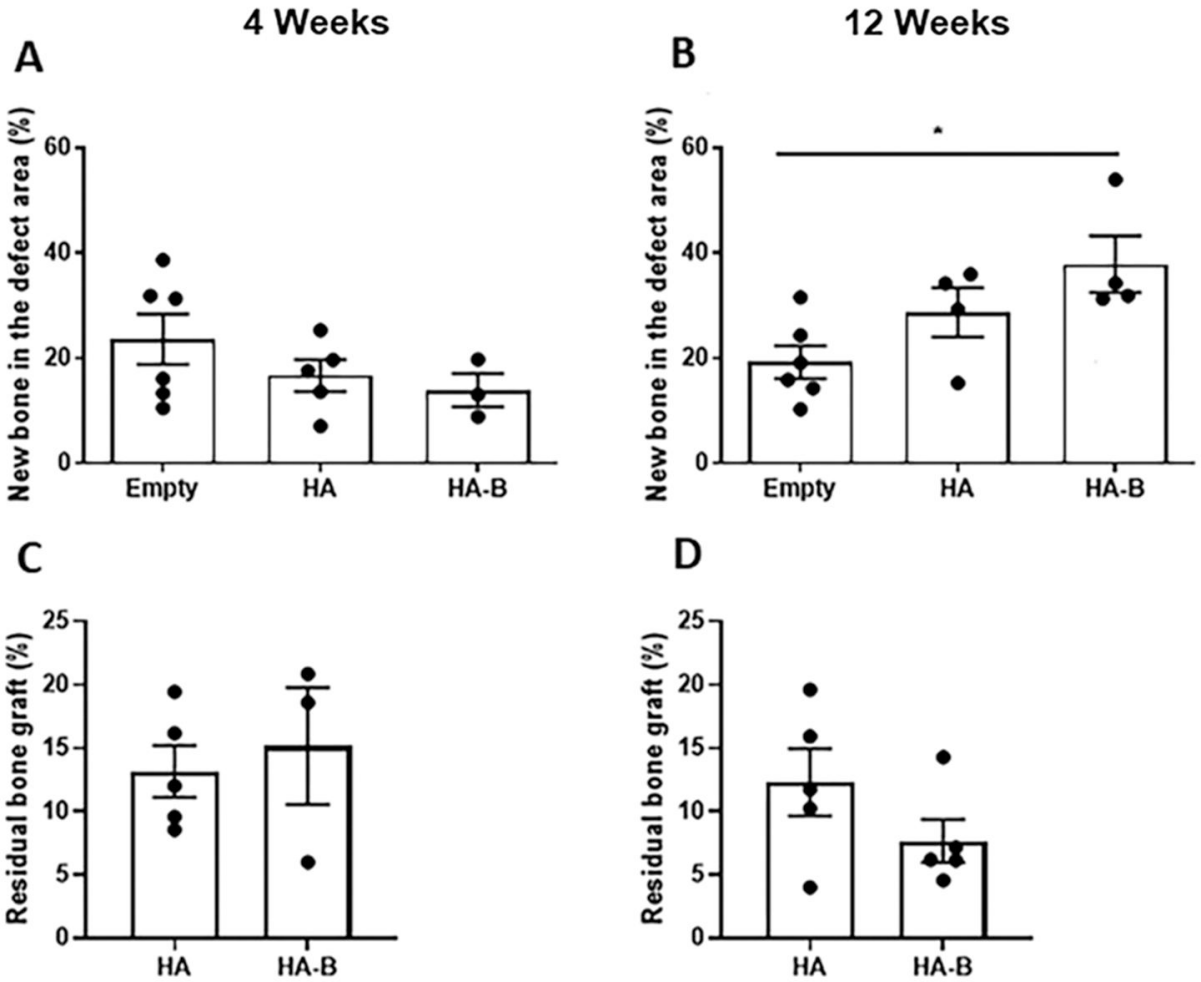
Quantitative analysis of new bone formation and residual bone graft in calvarial bone defects at 4 and 12 weeks post-implantation. Data are expressed as the percentage of new bone area and residual bone graft relative to the total defect area. For normally distributed data at 4 weeks (**A**) and (**C**), one-way ANOVA followed by Bonferroni’s multiple comparison test was used. For non-normally distributed data at 12 weeks (**B**) and (**D**), the Kruskal–Wallis test with Dunn’s post-hoc analysis was applied. *P* < 0.05 (*) was considered statistically significant.

Histomorphometric analysis revealed no significant differences in new bone formation among the groups at 4 weeks post-implantation (Figure 10A). By 12 weeks, the HA group showed a trend toward increased bone formation compared to the Empty control group, although this was not statistically significant. In contrast, the HA-B group demonstrated a significantly greater amount of new bone formation compared to the Empty control group (*P* < 0.05) (Figure 10B). The quantitative analysis of the residual bone graft was also performed to assess the *in vivo* resorption of the implanted materials. At 4 weeks, the percentage of residual graft was similar for the HA group and the HA-B group (Figure 10C). By 12 weeks, the HA group showed a minimal decrease in graft volume (Figure 10D). In contrast, the HA-B group exhibited a more pronounced decrease in residual material. However, no significant difference in the amount of residual bone graft between HA and HA-B was observed (*P* > 0.05).

### Push-out force evaluation

**Figure 11:**
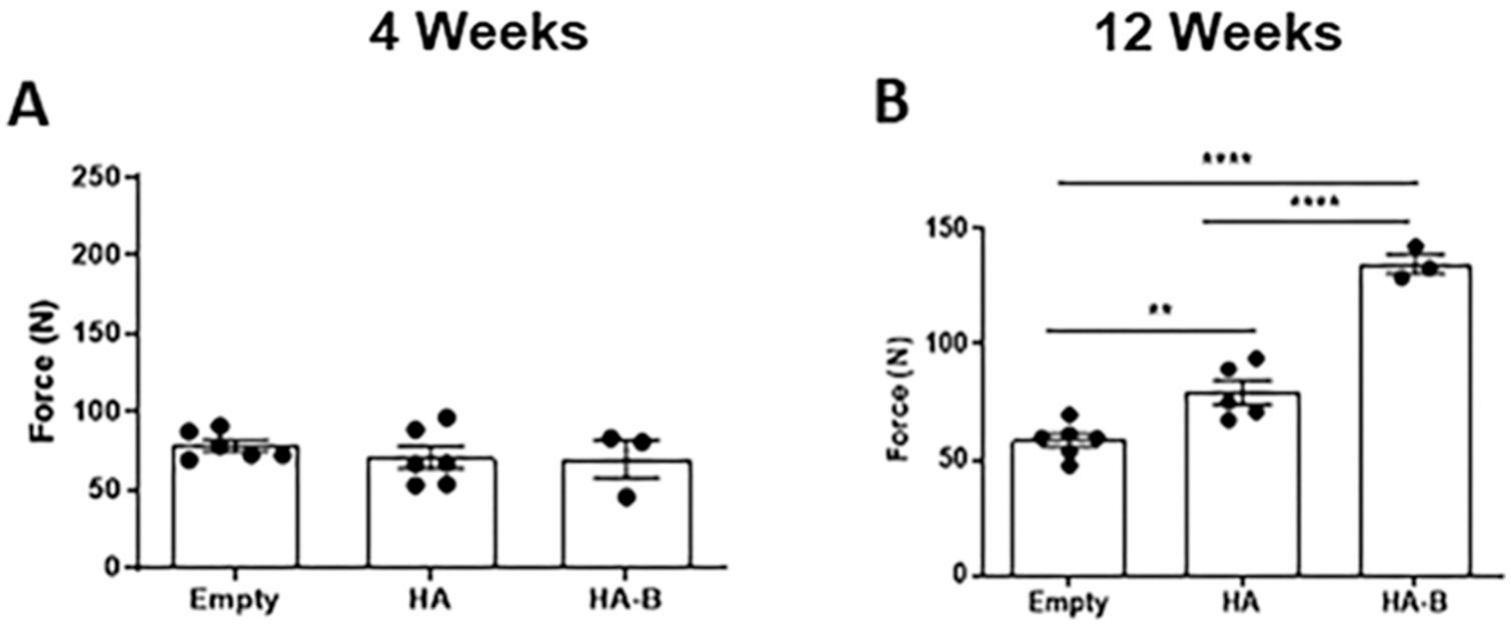
Push-out force measurements evaluating the interfacial strength between the regenerated tissue in the defect and host bone at 4 weeks (**A**) and 12 weeks (**B**) post-implantation. *P* < 0.01 (**), *P* < 0.0001 (****) were considered statistically significant.

Figure 11A and B present the push-out force measurements at 4 and 12 weeks post-implantation, respectively. At 4 weeks, no statistically significant differences were observed among the Empty, HA and HA-B groups. By 12 weeks, both the HA and HA-B groups demonstrated significantly greater push-out force than the Empty group, with the HA-B group also exhibiting a significantly higher value than the HA group.

## Discussion

This study shows that both HA and HA-B granules are biocompatible, osteoconductive, and capable of supporting bone regeneration without inducing systemic or local adverse effects. Qualitative histopathological evaluation of peri-implant tissues suggested a favorable tissue response with minimal inflammatory infiltration and no apparent tissue necrosis or severe foreign-body reaction. However, the present study was not designed to comprehensively assess biocompatibility at the immunological or molecular level, and further studies incorporating quantitative immunological and molecular inflammatory marker analyses are warranted to fully characterize the host response. Exogenous collagen sheets have been utilized to prevent fibrous tissue invasion [54]; however, the current study utilized the re-sutured pericranium as an autologous biological membrane. This approach ensured stabilization of the granular graft and provided a barrier to compartmentalize the bone healing area, allowing for a clear assessment of the regenerative potential of the binder-jet fabricated materials without the confounding influence of commercial membrane degradation products. These findings align with previous reports supporting the biological safety and regenerative potential of calcium phosphate materials in bone repair [6, 7, 55–57]. The high biocompatibility and safety observed across all groups are consistent with the complete removal of the water-soluble binders during processing. By ensuring the final granules are binder-free, the *in vivo* response is governed entirely by the osteoconductive HA and OCP mineral phases rather than residual processing chemicals.

While the current study evaluates the performance of binder-jet fabricated HA granules rather than porous structure-designed constructs or patient-specific scaffolds, this configuration was intentionally selected to enable evaluation of the intrinsic bioactivity of the fabricated material independent of scaffold architecture. At the same time, the findings highlight binder jetting as a versatile and scalable technique for producing standardized calcium phosphate graft materials with controlled composition and microstructure. These results provide a foundation for future investigations in which binder-jet technology, coupled with low-temperature phase transformation, may be applied to fabricate custom-shaped calcium phosphate scaffolds tailored to specific clinical defects. Such developments would further leverage the design flexibility of additive manufacturing to produce anatomically precise solutions for bone tissue engineering.

To gain insight into the cellular events underlying bone regeneration, histological sections were examined for the presence of bone-related cells, including osteoblasts, osteoclasts and osteocytes within the regenerated bone tissue of the defect sites across groups. Histological analysis revealed osteoblast presence in both HA and HA-B groups at 4 weeks, with significantly increased osteoblast numbers in the HA-B group at 12 weeks, indicating prolonged osteogenic activity likely stimulated by the OCP coating [58, 59]. The scarcity of osteoblasts in the Empty group underscores its inability to support active bone formation. This aligns with the illustrated H&E-stained histological images of calvarial bone defects in the Empty group and the nature of critical-sized defects, which fail to heal spontaneously without structural or biological support. Similarly, osteoclasts were observed in both HA and HA-B groups at 12 weeks, consistent with active bone remodeling. Their presence, particularly in the HA-B group, suggests that the OCP coating could further facilitate granule resorption and replacement by native bone through normal bone remodeling pathways [60, 61]. In contrast, a relative scarcity of osteoclasts was observed in the Empty group, suggesting limited bone remodeling activity in the absence of an implant. The number of osteocytes, mature bone cells embedded in the mineralized matrix, remained constant in all groups, reflecting similar maturation of the bone in all groups. The presence of osteocytes at the defect margins in the Empty group, together with the limited presence of osteoblasts and osteoclasts, suggests that bone healing has entered a relatively quiescent phase. This observation aligns with the concept that osteocytes are mature osteoblasts that have become embedded in the bone matrix [62]. It indicates that an initial stage of bone formation took place, during which osteoblasts produced new bone and some differentiated into osteocytes. However, the transient osteoblasts and osteoclasts are no longer active at the site [63]. This cellular pattern reflects a cessation in active bone remodeling, with the newly formed marginal bone progressing into a maintenance phase.

Despite no significant differences in the numbers of osteoblasts, osteoclasts and osteocytes between the HA-B and HA groups, bone formation tended to be greater in the HA-B group. This observation may indicate that the OCP coating influences the functional activity or maturation state of bone-forming and remodeling cells rather than their absolute number. Previous studies have suggested that OCP-containing materials can enhance osteoblast differentiation and modulate osteoclast-mediated remodeling, thereby supporting a balanced bone formation process [64]. In addition, OCP has been reported to influence extracellular matrix organization and mineralization and may interact with the local biological environment through ion release and surface-mediated signaling [65, 66]. Such mechanisms, including modulation of immune responses, angiogenesis, and progenitor cell recruitment, have been proposed in the literature for calcium phosphate biomaterials [67, 68]. However, these biological processes were not directly investigated in the present study and therefore, should be interpreted as potential mechanisms rather than confirmed effects.

Quantitative micro-CT analysis demonstrated significantly higher BV/TV in the HA-B group compared to HA and Empty at 12 weeks, reflecting enhanced bone formation consistent with histological observations; this improvement likely arises from the bioactive OCP coating, which stimulates osteoblast activity, local mineralization, and ion-mediated osteoinduction compared to uncoated HA or Empty defects. Interestingly, histomorphometric analysis did not show significant differences between HA and HA-B, although a similar trend was observed. This discrepancy likely arises from technical differences in how micro-CT assesses three-dimensional mineralized tissue across the entire defect, while histomorphometry evaluates two-dimensional tissue sections, which may miss localized variations. The higher sensitivity of volumetric micro-CT in this study is likely reinforced by the dynamic resorption of the HA-B. Since the HA-B group demonstrated a lower residual graft volume by 12 weeks compared to the HA group, the 3D analysis (BV/TV) is better positioned to capture the total volume of mineralized tissue replacing the resorbed granules across the entire 3D space. In contrast, 2D histological sections provide a localized view that may not fully reflect the extent of this volumetric substitution. Such inconsistencies have been reported in other studies and reinforce the importance of using complementary assessment techniques [69, 70].

Although enhanced bone formation was observed in defects treated with HA-B, the present study was not designed to directly investigate the biological mechanisms underlying this effect. Previous studies have suggested that calcium phosphate phases containing OCP may interact with the local biological environment during bone regeneration, including potential involvement of vascular-related processes. However, quantitative assessments of angiogenesis, such as immunohistochemical analysis of endothelial markers, were not performed in the current work. Therefore, the contribution of vascular-associated mechanisms to the observed enhancement of bone regeneration remains to be clarified and warrants further investigation in future studies. Despite differences in efficacy between implanted groups, both the HA and HA-B granules performed significantly better than the Empty group, which displayed only limited marginal bone formation and predominantly fibrous connective tissue at the defect center.

Push-out testing was utilized in this study to evaluate the mechanical integration between the regenerated tissue within the defect and the surrounding host bone [71, 72]. This method simulates the physiological forces that implants may encounter *in vivo* and provides a functional measure of the interfacial strength at the bone-implant interface. However, it should be noted that push-out testing primarily evaluates the interfacial bonding strength between the implant and surrounding bone rather than the overall biomechanical competence of the regenerated tissue. Consequently, the observed improvements should be interpreted as indicators of enhanced osseointegration and interfacial stability rather than direct evidence of compressive, tensile, or load-bearing mechanical capacity.

HA-B exhibited the highest push-out force at 12 weeks, significantly exceeding that of both the HA and Empty groups (*P* < 0.0001). This superior anchorage is likely attributed to the synergistic interplay of chemical and physical cues. Chemically, the intrinsic bioactivity of OCP facilitates its gradual conversion *in vivo* into bone-like apatite, potentially accelerating the formation of a direct, chemical bond with native bone tissue [68, 73, 74]. Beyond surface bonding, our results suggest a dynamic relationship between granule resorption and bone regeneration. Quantitative analysis of the residual bone graft (Figure 10C and D) revealed that the HA-B group underwent a notable reduction in material volume from 4 weeks to 12 weeks, whereas the HA group remained relatively constant. The resorption of the OCP coating likely plays a dual role in enhancing integration. First, it stimulates localized bone remodeling through the release of calcium and phosphate ions, which are known to recruit and activate osteoblasts. Second, this resorption facilitates spatial remodeling by gradually increasing the available space within the defect site. Unlike the relatively biostable HA substrate, the OCP coating undergoes targeted degradation, creating micro-architectural voids that allow for the deeper ingrowth of mineralized tissue. This coordinated space-making and bone-filling process leads to a more integrated and continuous bone-granule bridge across the central defect, transforming the interface into a structurally unified, load-bearing composite. In contrast, the Empty group’s measurable load resulted only from marginal bone formation at the edges, leaving the central defect unhealed, as confirmed by histological and micro-CT analyses.

Physically, the microstructure of the coating likely plays a pivotal role in the observed bone regeneration. The transition from the acicular HA to the foliated, leaf-like OCP crystals (Figure 4E and G) creates a highly complex, biomimetic nano-topography. Due to the shared precipitation-based nature of both phases, the OCP layer is epitaxially integrated into the HA substrate, precluding the measurement of a discrete coating thickness. This observation is consistent with our previous work on biomimetic OCP coatings, where similar growth kinetics resulted in a layer thickness of approximately 4.3–8.7 µm [52]. This leaf-like crystal morphology may increase the available surface area for the adsorption of cell-adhesive proteins such as fibronectin and vitronectin from the blood clot, which in turn provides a more favorable environment for initial cell attachment [75, 76]. The interconnected valleys between the leaf-like crystals may also provide anchoring sites for osteoblast filopodia, triggering mechanotransduction pathways that accelerate early osseointegration. While further studies involving quantitative surface analysis are needed to isolate these effects, our results suggest that the OCP coating could provide both chemical and topographical cues to the healing bone tissue. This emphasizes the importance of interpreting push-out results in conjunction with biological assessments, as mechanical measurements alone may not fully reflect the extent of new bone formation. Nevertheless, the push-out data also demonstrate that bone regenerated with binder-jet fabricated granules, particularly those coated with biomimetic OCP, can achieve a level of mechanical integration by creating a structurally unified, load-bearing composite that is comparable to or exceeds that of bone formed by natural healing without the aid of implants.

The findings of this *in vivo* study are in good agreement with our previous *in vitro* investigation using human bone marrow and umbilical cord-derived stem cells, which demonstrated that binder-jet fabricated HA and biomimetic OCP-coated binder-jet fabricated HA supported cell attachment, proliferation and osteogenic differentiation [43]. In that study, both materials supported upregulation of osteogenic markers (ALP, RUNX-2, osterix (OSX), and osteocalcin (OCN)) and mineralized matrix deposition. These *in vitro* results are corroborated by the increased new bone formation and superior mechanical integration observed in the OCP-coated group *in vivo*. Together, these complementary studies support the potential of binder-jet fabricated HA and biomimetic OCP-coated binder-jet fabricated HA for bone regeneration applications.

This study has several limitations. First, the rat calvarial defect represents a non-load-bearing model, which may not fully replicate clinical scenarios involving load-bearing bones, and the mechanical evaluation relied primarily on push-out testing, which mainly assesses the interfacial bonding strength between the implant and surrounding bone. As such, it does not fully represent the overall biomechanical competence of the regenerated tissue or the complex compressive, tensile and torsional forces encountered in clinical load-bearing bone repair. Second, although the re-sutured pericranium served as a natural biological barrier to prevent soft tissue invasion into the defect, a commercial membrane commonly used in guided bone regeneration was not employed. Therefore, the regenerative outcomes reflect the intrinsic performance of the implanted materials under a biological barrier condition rather than a membrane-assisted guided bone regeneration (GBR) model. Third, the biological mechanisms underlying the enhanced bone regeneration observed with biomimetic OCP-coated binder-jet fabricated HA granules were not directly investigated. Although our previous *in vitro* studies have demonstrated osteogenic cellular responses to these materials [43], the present study did not evaluate molecular or cellular pathways associated with bone regeneration in the *in vivo* model. In particular, markers related to osteogenesis, osteoimmune regulation (e.g. cytokine profiles and macrophage polarization) and angiogenesis were not examined. Therefore, the underlying regenerative mechanisms responsible for the observed bone formation could not be fully elucidated and should be considered potential mechanisms inferred from prior literature rather than confirmed effects in the current study. Fourth, the evaluation of bone quality was limited to volumetric micro-CT and histological analyses. Additional micro-CT parameters, such as bone mineral density and trabecular microarchitecture, were not assessed. Fifth, although distinct topographical differences between acicular HA and foliated OCP crystals were observed, detailed quantitative surface characterization was not performed. Their quantification would further isolate the role of topographical cues in the observed osseointegration. Sixth, the optimization of the OCP coating content was not investigated, despite previous reports indicating that the osteogenic effect of OCP may be dose-dependent [35, 36]. Finally, although the materials were fabricated using binder-jet, they were evaluated in granular form, which does not fully exploit the architectural design flexibility of additive manufacturing for producing patient-specific scaffolds with controlled macroporous structures. Future studies should therefore incorporate load-bearing animal models, mechanistic analyses of osteogenic and osteoimmune pathways, angiogenesis-related assessments, quantitative surface characterization, and dose-dependent optimization of OCP coatings, as well as architecturally optimized 3D-printed scaffolds to further clarify the role of OCP in bone regeneration and strengthen the translational relevance of these findings.

## Conclusion

Binder-jet fabricated HA, and biomimetic OCP-coated binder-jet fabricated HA granules both demonstrated the ability to support bone regeneration in a critical-sized rat calvarial defect model. The results further showed that the biomimetic OCP coating enhanced new bone formation and improved integration with host tissue compared with uncoated HA. These findings confirm the bone regenerative potential of binder-jet fabricated HA constructs and indicate that biomimetic OCP surface modification can further improve their biological performance. Overall, the combination of binder-jet fabricated and biomimetic OCP coating represents a promising strategy for developing bioactive calcium phosphate scaffolds for bone repair.

## Supplementary Material

rbag076_Supplementary_Data

## Data Availability

The datasets used and/or analyzed during the current study are available from the corresponding author on reasonable request.
