# Supplementary material for: Bone regenerative efficacy of binder-jet fabricated hydroxyapatite granules with and without biomimetic octacalcium phosphate-coated modification in a rat critical-sized calvarial defect model
Source: Regen Biomater. 2026 Apr 20;13:rbag076. doi: 10.1093/rb/rbag076 (PMC13198882; doi:10.1093/rb/rbag076)

**Supporting information**

**S1 Fig.** Representative photographs of surgical procedures of sample implantation in a rat calvarial bone defect model. (A) Anesthesia & intubation (B) Skin incision & exposure of periosteum. (C) Trephine drilling (D) Creation of bilateral critical-sized, full-thickness calvarial bone defects (5 mm in diameter). (E and F) Sample implantation and defect location. (G) Wound closure by suturing the connective tissue and pericranium to secure the implants. (H) Skin closure using 4-0 non-absorbable silk sutures.


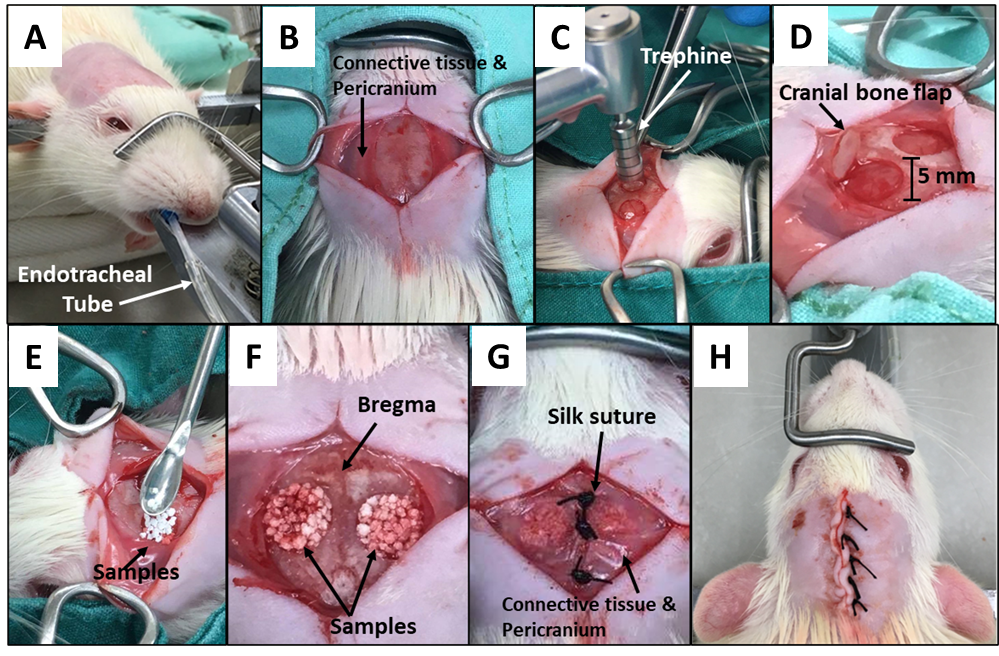

Supplement: rbag076_Supplementary_Data [file rbag076_supplementary_data.zip › Supplementary Figure 1.docx]
