# Supplementary material for: Bone regenerative efficacy of binder-jet fabricated hydroxyapatite granules with and without biomimetic octacalcium phosphate-coated modification in a rat critical-sized calvarial defect model
Source: Regen Biomater. 2026 Apr 20;13:rbag076. doi: 10.1093/rb/rbag076 (PMC13198882; doi:10.1093/rb/rbag076)
Supplement: rbag076_Supplementary_Data [file rbag076_supplementary_data.zip › Supplementary Figure 2.docx]

**Supporting information**

**S2 Fig. The rat body weight gain over 4- and 12-weeks post-implantation in all experimental groups**
