# Supplementary material for: Bone regenerative efficacy of binder-jet fabricated hydroxyapatite granules with and without biomimetic octacalcium phosphate-coated modification in a rat critical-sized calvarial defect model
Source: Regen Biomater. 2026 Apr 20;13:rbag076. doi: 10.1093/rb/rbag076 (PMC13198882; doi:10.1093/rb/rbag076)
Supplement: rbag076_Supplementary_Data [file rbag076_supplementary_data.zip › Supplementary Table 1.docx]

**Supporting information**

**S1 Table. Blood biochemistry parameters: pre-implantation and post-implantation of 3D Printed HA samples. Data are presented as mean ± standard error of the mean (SEM).**

|  | **4 weeks** | | **12weeks** | |
| --- | --- | --- | --- | --- |
| **Experimental Groups** | **ALT (U/L)** | **Creatinine (mg/dL)** | **ALT (U/L)** | **Creatinine (mg/dL)** |
| Pre-implantation | 49.00 ± 1.64 | 0.68 ± 0.02 | 43.44 ± 1.59 | 0.62 ± 0.01 |
| Post-implantation | 42.28 ± 1.25 | 0.77 ± 0.03 | 52.50 ± 2.07 | 0.82 ± 0.02 |
